# Editorial: Cancer-related hypercalcemia and potential treatments

**DOI:** 10.3389/fendo.2023.1281731

**Published:** 2023-09-12

**Authors:** Lorenzo Scappaticcio, Arif Nur Muhammad Ansori, Pierpaolo Trimboli

**Affiliations:** ^1^ Unit of Endocrinology and Metabolic Diseases, AOU University of Campania “Luigi Vanvitelli”, Naples, Italy; ^2^ Department of Advanced Medical and Surgical Sciences, University of Campania “Luigi Vanvitelli”, Naples, Italy; ^3^ Faculty of Science and Technology, Universitas Airlangga, Surabaya, Indonesia; ^4^ Uttaranchal Institute of Pharmaceutical Sciences, Uttaranchal University, Dehradun, India; ^5^ Clinic of Endocrinology and Diabetology, Lugano and Mendrisio Regional Hospital, Ente Ospedaliero Cantonale, Bellinzona, Switzerland; ^6^ Faculty of Biomedical Sciences, Università della Svizzera Italiana, Lugano, Switzerland

**Keywords:** cancer-related hypercalcemia, parathyroid carcinoma, denosumab, cinacalcet, somatostatin analogues

The Research Topic “*Cancer-related hypercalcemia and potential treatments*” aimed to collect new advances on the management of hypercalcemia associated with malignancy. The call for submission started at the beginning of 2022 and it was closed on May 2023, finally including six papers written and reviewed by international experts in this topic (Almuradova and Cicin, Farooki et al., Roukain et al., Su et al., Herrera-Martínez et al., Sapuppo et al.).

Cancer-related hypercalcemia (CrH), also known as hypercalcemia of malignancy (HCM), is the most common metabolic complication of malignancies affecting between 2% and 30% of patients with cancer, with rates that vary depending on cancer type and disease stage (1). Hypercalcemia in the cancer setting can be the flag of poor health outcomes and high mortality risk. However, thanks to the advent of more effective antineoplastic and supportive measures, the 30-day mortality rates of 50% in patients with CrH reported in the early series has greatly decreased (2). In fact, nowadays it is estimated that CrH is associated with an in-hospital mortality of 6.8% (3). It is therefore easy to understand that a large effort by worldwide clinical experts has recently made to improve fast the morbidity and mortality of patients with CrH. Moreover, it is no coincidence that this Frontiers in Endocrinology Research Topic and the first Endocrine Society clinical practice guideline on the treatment of HCM in adults have been conceived around the same time. The latter means that hypercalcemia in the cancer setting warranted international research attention and evidence-based recommendations.

Almuradova and Cicin reviewed the current literature on the mechanisms, diagnosis and management of malignant hypercalcemia. Whatever the cause of CrH, timely and efficacious treatment to control hypercalcemia is critical to minimize morbidity and time hospitalized (Almuradova and Cicin). The mainstay of therapy is represented by intravenous (IV) saline hydration and antiresorptive agents (i.e., bisphosphonates and/or denosumab) (Almuradova and Cicin). In severe CrH [i.e., serum calcium >14 mg/dL (3.5 mmol/L)], intramuscular or subcutaneous calcitonin should be added as the initial treatment, considering the faster onset of action than antiresorptive agents (i.e., 4-6 hours compared to 2-3 days of bisphosphonates and 3-10 days of denosumab) (Almuradova and Cicin). However, calcitonin use should be limited to 48 to 72 hours due to tachyphylaxis (Almuradova and Cicin). In refractory/recurrent CrH on bisphosphonate therapy, denosumab should be started (Almuradova and Cicin, Farooki et al., Roukain et al.). Other treatment options for refractory/recurrent CrH are the following: oral phosphorus to treat severe hypophosphatemia that occurs frequently in humoral (i.e., parathyroid hormone–related peptide) CrH, since it binds calcium and then, in the presence of normal renal function, is eliminated and also can be deposited in bone or possibly in extra skeletal tissues (Farooki et al.); corticosteroids work to decrease calcium likely *via* multiple mechanisms, and are highly effective in CrH characterized by calcitriol levels (Almuradova and Cicin, Farooki et al.); furosemide is suggested only in a well hydrated hypercalcemic patient receiving normal saline to transiently determine small reductions of serum calcium (Almuradova and Cicin, Farooki et al.). When compared with denosumab, for bisphosphonates dose adjustment and longer administration time (i.e., 30 to 60 minutes) are required in case of underlying renal insufficiency to prevent acute-phase response and worsening renal function (Almuradova and Cicin, Farooki et al.). Furthermore, particularly when parenteral bisphosphonates and denosumab are used at an oncologic dosing frequency (monthly to every 3 months), rare but significant adverse effects, such as osteonecrosis of the jaw and atypical femur fracture, can occur (Almuradova and Cicin, Farooki et al.).

In case of CrH due to parathyroid carcinoma (PC), the first-line treatment consists of surgical en bloc resection together with the ipsilateral thyroid lobe, even in the presence of metastatic disease, once control of severe hypercalcemia has been achieved (Roukain et al.). The reduction of moderate to severe hypercalcemia due to PC usually requires IV bisphosphonates or denosumab as the initial therapy (Almuradova and Cicin, Roukain et al.); conversely, the calcimimetic cinacalcet is started in case of mild hypercalcemia (Almuradova and Cicin, Roukain et al.). These are practical aspects regarding the use of cinacalcet in CrH due to PC, since it often is poorly tolerated due to its gastrointestinal side effects (i.e., nausea and vomiting), which are usually dose-dependent, and it has longer onset of action (i.e., 2-3 days) than bisphosphonates. In this Research Topic there is the paper by Su et al., which is the first systematic review on diagnosis and treatment of liver metastases of PC published to date. Approximately 25%–30% of patients with PC develop distant metastases at some point in the course of the disease. The most common sites of distant metastases are the lungs (40%) and liver (10%), but PC can also spread to the abdominal lymph nodes, bones, pleura, pericardium, and pancreas in rare cases. Since most liver metastases of PC are functional, diagnosis should be suspected when serum PTH and calcium increase. Enhanced computed tomography (CT) and magnetic resonance imaging (MRI) can provide valuable support for the diagnosis of liver metastases, but whether 18F-FDG Positron emission tomography (PET)/CT, 18F-FCH PET/CT, or 11C-choline PET/CT can be used as a diagnostic tool this requires further studies. Therapy of liver metastases by PC is palliative and primarily aims to improve symptoms of hypercalcemia through surgery and/or ablation therapies.

When CrH is associated with neuroendocrine tumors (NETs), somatostatin analogues (SSAs) can serve as a further therapy to reduce hypercalcemia, as reported by two case reports in our Research Topic (Herrera-Martínez et al., Sapuppo et al.). The beneficial effect on hypercalcemia of SSAs is consequence of multiple actions, both indirect (i.e., tumor volume reduction) and direct (i.e., reduction of PTH secretion due to somatostatin receptor expression on parathyroid cells) (Herrera-Martínez et al., Sapuppo et al.).

**Table 1** summarizes the main findings of each paper included in this Research Topic.

**Table 1:** Main findings of each paper included in this Research Topic.

Main findings	Reference
The mainstay of therapy is represented by IV saline hydration and antiresorptive agents	Almuradova and Cicin
In refractory/recurrent CrH on bisphosphonate therapy denosumab should be started. Other treatment options are oral phosphorus, corticosteroids, furosemide	Farooki et al.
In case of CrH due to PC, in moderate to severe hypercalcemia IV bisphosphonates or denosumab are the initial therapy; conversely, cinacalcet is started in mild hypercalcemia	Roukain et al.
Therapy of liver metastases by PC is palliative and primarily aims to improve symptoms of hypercalcemia through surgery and/or ablation therapies	Su et al.
When CrH is associated with NETs, SSAs can serve as a further therapy to reduce hypercalcemia	Herrera-Martínez et al., Sapuppo et al.

IV, intravenous; CrH, cancer-related hypercalcemia; PC, parathyroid carcinoma; NETs, neuroendocrine tumors; SSAs, somatostatin analogues.

We enjoyed hosting this exciting Research Topic and we thank all the authors for their excellent contributions.

## Author contributions

LS: Conceptualization, Writing – original draft. AA: Writing – review & editing. PT: Writing – review & editing.
